# Deciphering the Dichotomous Effects of PGC-1α on Tumorigenesis and Metastasis

**DOI:** 10.3389/fonc.2018.00075

**Published:** 2018-03-23

**Authors:** Simon-Pierre Gravel

**Affiliations:** Laboratory of Metabolic Immunopharmacology, Faculty of Pharmacy, University of Montreal, Montreal, QC, Canada

**Keywords:** PGC-1, cancer, metastasis, OXPHOS, oncogenes, tumor suppressors, microenvironment, reactive oxygen species

## Abstract

Metabolic reprogramming confers cancer cells the ability to grow and survive under nutrient-depleted or stressful microenvironments. The amplification of oncogenes, the loss of tumor suppressors, as well as context- and lineage-specific determinants can converge and profoundly affect the metabolic status of cancer cells. Cumulating evidences suggest that highly glycolytic cells under the influence of oncogenes such as BRAF, or evolving in hypoxic microenvironments, will promote metastasis through modulation of multiple steps of tumorigenesis such as the epithelial-to-mesenchymal transition (EMT). On the contrary, increased reliance on mitochondrial respiration is associated with hyperplasic rather than metastatic disease. The PGC-1α transcriptional coactivator, a master regulator of mitochondrial biogenesis, has recently been shown to exert antimetastatic effects in cancer, notably through inhibition of EMT. Besides, PGC-1α has the opposite role in specific cancer subtypes, in which it appears to provide growth advantages. Thus, the regulation and role of PGC-1α in cancer is not univocal, and its use as a prognostic marker appears limited given its highly dynamic nature and its multifaceted regulation by transcriptional and posttranslational mechanisms. Herein, we expose key oncogenic and lineage-specific modules that finely regulate PGC-1α to promote or dampen the metastatic process. We propose a unifying model based on the systematic analysis of its controversial implication in cancer from cell proliferation to EMT and metastasis. This short review will provide a good understanding of current challenges associated with the study of PGC-1α.

## Introduction

Deregulated metabolism is now a well-documented hallmark of cancer cells that provides them with the ability to grow and survive under nutrient-depleted or stressful microenvironments (1, 2). Metabolic reprogramming is associated with important aspects of oncogenesis such as the epithelial-to-mesenchymal transition (EMT), a complex process that allows cancer cells to invade surrounding tissues and migrate to the vasculature (3–8). Oncogenes such as BRAF and MYC, through multifaceted shaping of central carbon metabolism and mitochondrial activity, can dictate the propensity of cancer cells to rely on specific metabolic routes such as glycolysis and glutaminolysis, thus revealing susceptibilities to metabolic drugs (9–13). While a preferential reliance on aerobic glycolysis (Warburg effect) instead of mitochondrial respiration has long been linked to increased tumor burden and poor outcome, it is now recognized that mitochondria have additional key roles in tumorigenesis and represent potential therapeutic targets (14–16).

Mitochondrial biogenesis and function are under the tight control of the peroxisome proliferator-activated receptor-ϒ coactivator-1 (PGC-1) family of transcriptional coactivators, among which, PGC-1α has been the most extensively studied member (17, 18). PGC-1α exerts its function through molecular scaffolding of diverse members of the nuclear receptors family, transcription factors, the TRAP/mediator complex, as well as various acetyl/methyltransferase complexes (19, 20). The magnitude and specificity of PGC-1α-directed transcriptional programs can be achieved through at least three distinct mechanisms: (1) by modulation of its expression level, thus limiting the recruitment of binding partners, (2) by the availability and functional status of its binding partners, and (3) by posttranslational modifications, which can modify both its expression level and the nature of its interactions (21–23). Hence, the complex regulation of PGC-1α is function of the cellular landscape associated with a given context, which makes this coactivator a master integrator of the nutritional and redox statuses, cellular stress, infection, and oncogenic signaling. Given its intrinsic capacities to orchestrate cellular bioenergetics, it is not surprising that PGC-1α is a key player in a variety of systems including muscular physiology, aging, and neurological functions (24, 25).

Besides its well-established functions as a master regulator of mitochondrial biogenesis and respiration, PGC-1α has been shown to regulate many other processes that could be linked to oncogenesis. First, PGC-1α conjointly promotes the expression of antioxidant genes that will protect cells from the deleterious effects of reactive oxygen species (ROS) as byproducts of the electron transport chain (26, 27). Second, PGC-1α can modulate the expression of VEGF, a key player in inflammation and angiogenesis (28). While promoting catabolism of glucose and fatty acids in line with increased mitochondrial mass, PGC-1α can also promote gluconeogenesis and lipogenesis, and thus could exert opposite anabolic functions (29–32). This apparent contradiction in PGC-1α functions, which could be explained by context-specific determinants, is key to the understanding of its dichotomous effects on cancer development. Although PGC-1α could be considered at first as a potential tumor suppressor by promoting mitochondrial oxidation and opposing aerobic glycolysis, recent studies revealed that its role in tumorigenesis and metastasis is not univocal. In the following sections, we will present evidences associating both pro- and anti-tumorigenic roles of PGC-1α and will provide a unifying model based on multifactorial determinants that shape its role in cancer.

## Dichotomous Effects of PGC-1α on Oncogenesis

### PGC-1α as a Prognostic Marker

**Figure 1:**
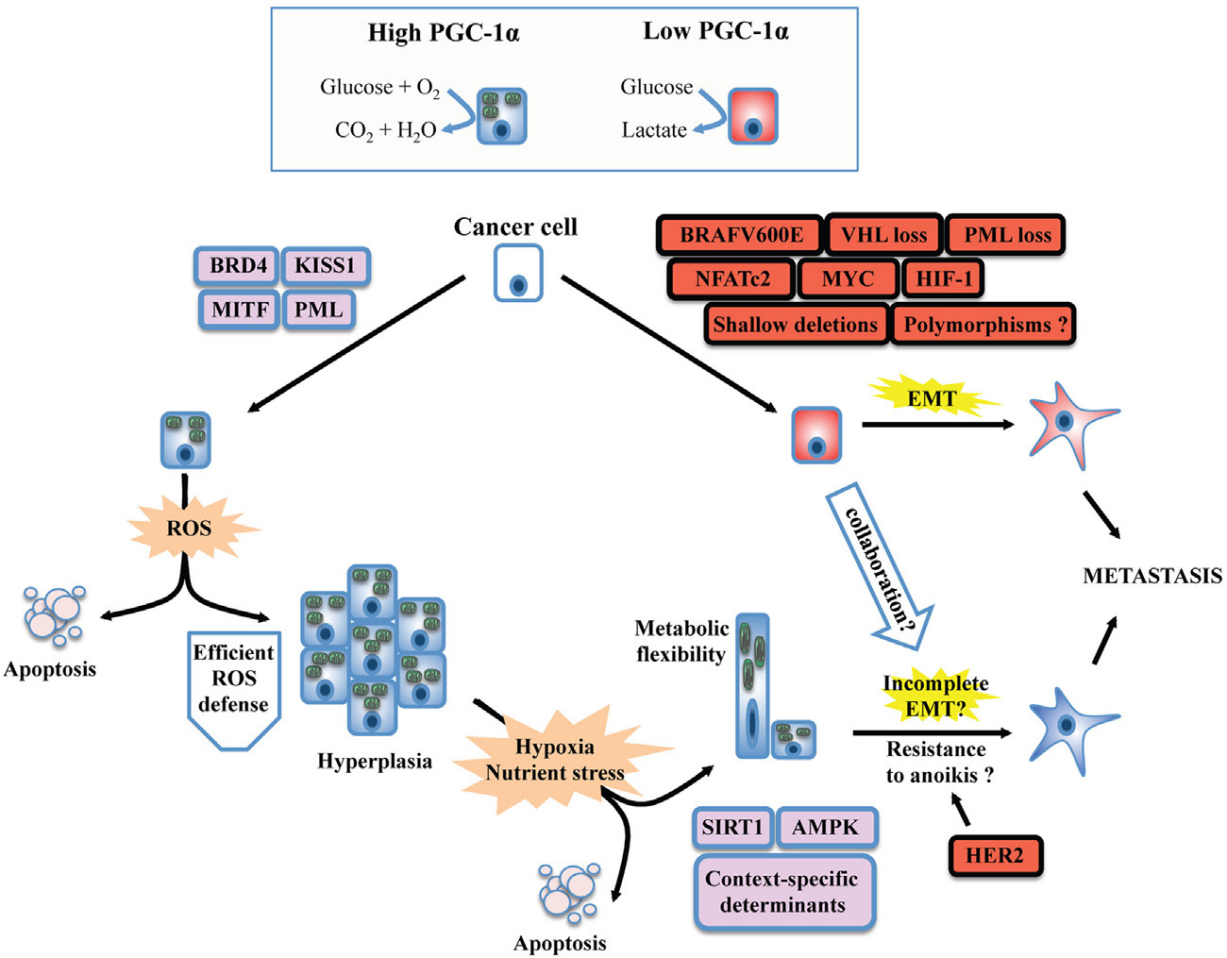
Unifying model for the dichotomous effects of PGC-1α on tumorigenesis and metastasis. PGC-1α status usually reflects the metabolic profile of cancer cells. High PGC-1α will promote mitochondrial metabolism and OXPHOS, while low PGC-1α is usually associated with increased glycolysis. The switch between glycolytic and OXPHOS status will be the result of a complex interplay between oncogenes, tumor suppressor, and context-dependent determinants such as lineage-specific proteins. BRAF mutants, MYC amplification, and the loss of tumor suppressors such as VHL will downregulate PGC-1α and will confer a glycolytic phenotype in line with the establishment of the epithelial-to-mesenchymal transition (EMT). Besides, context-specific proteins such as microphtalmia-associated transcription factor (MITF, melanocyte lineage) or KISS1 will promote *PPARGC1A* transcription or PGC-1α protein stabilization, respectively. The emergence of metastasis in situations of high PGC-1α despite its inhibitory role on EMT suggests that PGC-1α will confer selective advantage to cancer cells evolving in specific contexts. Increased OXPHOS due to PGC-1α activity will generate reactive oxygen species (ROS). To overcome this stress, an efficient antioxidant gene expression program must be induced, which will depend on the activity of PGC-1α itself, the functionality, and availability of its transcriptional partners, as well as other context-dependent regulators. High PGC-1α has been linked to increased tumor growth (hyperplasia), but not necessarily to promote metastasis. The selective pressure imposed to cancer cells by the tumor microenvironment will favor the survival of well-adapted cells, likely through the regulation of PGC-1α by SIRT1 and AMP kinase (AMPK), as well as other context-specific factors. Notably, high PGC-1α expression in specific cancer cells can generate metabolic hybrids (both OXPHOS and glycolytic), making these cells particularly flexible. Given that metastatic and circulating tumor cells with high PGC-1α levels have been detected, several mechanisms could explain the propensity of some high PGC-1α cells to metastasize. First, the completeness of EMT is not mandatory. Second, context-specific oncogenes such as the HER2 receptors could increase survival upon cellular matrix detachment (anoikis). Third, glycolytic cells (low PGC-1α) could collaborate in the migration and invasion process of high PGC-1α cells through the release of tissue remodeling factors. Finally, the growth advantages conferred by PGC-1α might increase cancer cell survival and growth at distant metastatic niches, in spite of their low metastatic potential.

The association between PGC-1α levels and disease-free survival in humans revealed that its sole expression is not necessarily predictive of outcome (33). While low PGC-1α levels have been associated with poor outcome in breast, prostate, and VHL-deficient clear renal cell carcinoma (34–36), high PGC-1α levels have also been associated with poor outcome in the context of melanoma, breast, and prostate cancer (37–40), revealing that other factors such as tumor heterogeneity and context-specific transcriptional programs are critical determinant of the implication of PGC-1α in disease progression. Importantly, PGC-1α levels do not necessarily follow the ones of OXPHOS genes in disease progression (41), suggesting that its regulation through posttranslational modification is underevaluated in the context of cancer research. There is currently limited evidence that genomic alterations of the *PPARGC1A* gene can be associated with disease outcome. Mutations in the *PPARGC1A* gene have been detected (42, 43), a specific polymorphism (Thr612Met) has been associated with some classes of breast cancer (44), and numerous polymorphisms have been linked to ovarian cancer susceptibility (45). While *PPARGC1A* amplification appears to be a rare event (38), important shallow deletions of *PPARGC1A* have been found in metastasis derived from prostate cancer in human studies (36). This intriguing observation suggests that selective pressure can diminish PGC-1α levels, which could confer specific advantages to subsets of cells through the course of tumor evolution. Taken together, these studies suggest that the contribution of PGC-1α on disease progression will be function of tumor history and cancer subtypes, and thus do not support its sole use as a prognostic marker for cancer medicine. In order to get a better understanding of the dualistic nature of PGC-1α in cancer development, we surveyed the literature and evaluated the impact of PGC-1α experimental manipulation on key aspects of disease progression such as cancer cell proliferation, primary tumor growth, ROS detoxification, the modulation of EMT, and metastasis. From these studies, it can be concluded that both high and low PGC-1α levels in primary tumor can potentially lead to metastatic disease, which can be easily explained by the oncogenic landscape and context-specific determinants within the primary tumor (Figure 1).

### PGC-1α and Mitochondrial Function

Strikingly, independently of the final outcome that PGC-1α has on oncogenesis, all examined studies fully support its classic role as a positive regulator of mitochondrial biogenesis and mitochondrial respiration in cancer cells (31, 35, 38–40, 46–54). This suggests that the dichotomous aspect of PGC-1α on cancer must take root in other aspects of cancer development that could be differentially regulated by PGC-1α. These aspects will be developed in the following sections.

### Cellular Growth Is Conditioned by the Microenvironment

Opposite to its uniform impact on mitochondrial function, PGC-1α has divergent outcomes on cell proliferation and tumor growth, which can be largely explained by cancer type. For example, elevated PGC-1α expression impairs clear renal cell carcinoma and prostate cancer cell lines proliferation and their ability to form tumors (35, 36), while it does the inverse in a subset of melanoma cells (38). Besides, the impact of PGC-1α expression on cancer cell proliferation does not necessarily correlate with the growth of primary tumors established with the same cell lines (31, 50, 55). An explanation for these discrepancies is that *in vitro* culture conditions are not representative of the microenvironment experienced by cancer cells *in vivo*, which suggests that elevated PGC-1α expression might provide growth advantage *in vivo*, notably by increasing glucose uptake or by promoting the use of glutamine to support mitochondrial respiration and lipogenesis (31, 32, 50). Interestingly, a recent study revealed that PGC-1α potentiates bioenergetics capacity and fuel flexibility of cancer cells (56). Thus, the development of appropriate *in vitro* culture conditions that better mimic the tumor microenvironment will be required to further determine which metabolic processes are selectively modulated by PGC-1α to confer growth advantage (57).

### Cell Survival Is Function of ROS Detoxification

In specific models, the growth-suppressive effects of PGC-1α could be attributed to its capacity to induce apoptosis (47, 48, 58), to increase ROS production, and DNA damage response (35), and to block cell cycle progression (36). One explanation for these unexpected findings is that PGC-1α overexpression can lead to dramatic imbalance between ROS production by the electron transport chain and ROS detoxification, both orchestrated by PGC-1α (27). Some cell lines might respond well to increased PGC-1α input, while others might be unable to mount a proper antioxidant gene program due to differential availability of transcriptional partners or effectors. Only one study systematically measured the impact of PGC-1α overexpression and knockdown on a panel of antioxidant genes, confirming that it can indeed promote ROS detoxification in a subset of melanoma cells (38).

### OXPHOS and Glycolysis Are Modulators of EMT

A novel link between PGC-1α and the inhibition of the EMT gene expression program is emerging and supports its tumor suppressive functions. Indeed, PGC-1α has been shown to positively regulate the expression of the epidermal marker E-cadherin and inhibit the expression of multiple genes within the integrin/TGFβ/WNT pathways (49, 59–61). There is now strong evidence that metabolic changes associated with cancer, such as mitochondrial dysfunction and increased glycolysis, efficiently promote EMT activation (6), which is in agreement with the notion that PGC-1α antagonizes EMT. Moreover, OXPHOS downregulation is associated with low survival in most human cancers and shows negative correlation with EMT (41). To reconcile these facts with the observation that PGC-1α can indeed promote metastasis (39, 56), context-specific and spatiotemporal regulation of PGC-1α must be taken into account. Notably, tumor cells can exhibit wide differences in the completeness of their EMT program and still be able to invade (62). Also, PGC-1α expression and functional status (acetylation, phosphorylation) is likely highly heterogeneous within a given tumor according to its highly dynamic expression (61). Thus, its inhibitory effect on EMT might be transient, bypassed by context-specific oncogenes, or influenced by neighbor cells. It will be important to determine how cells expressing high and low levels of PGC-1α can coexist within a tumor and if they can collaborate during EMT. Finally, very limited studies systematically evaluated the impact of PGC-1α on VEGF production, angiogenesis, and anchorage-independent growth. Therefore, it would be too early to conclude that PGC-1α can indeed directly regulates these important tumorigenesis steps.

## Oncogenic and Lineage-Specific Regulation of PGC-1α

**Figure 2:**
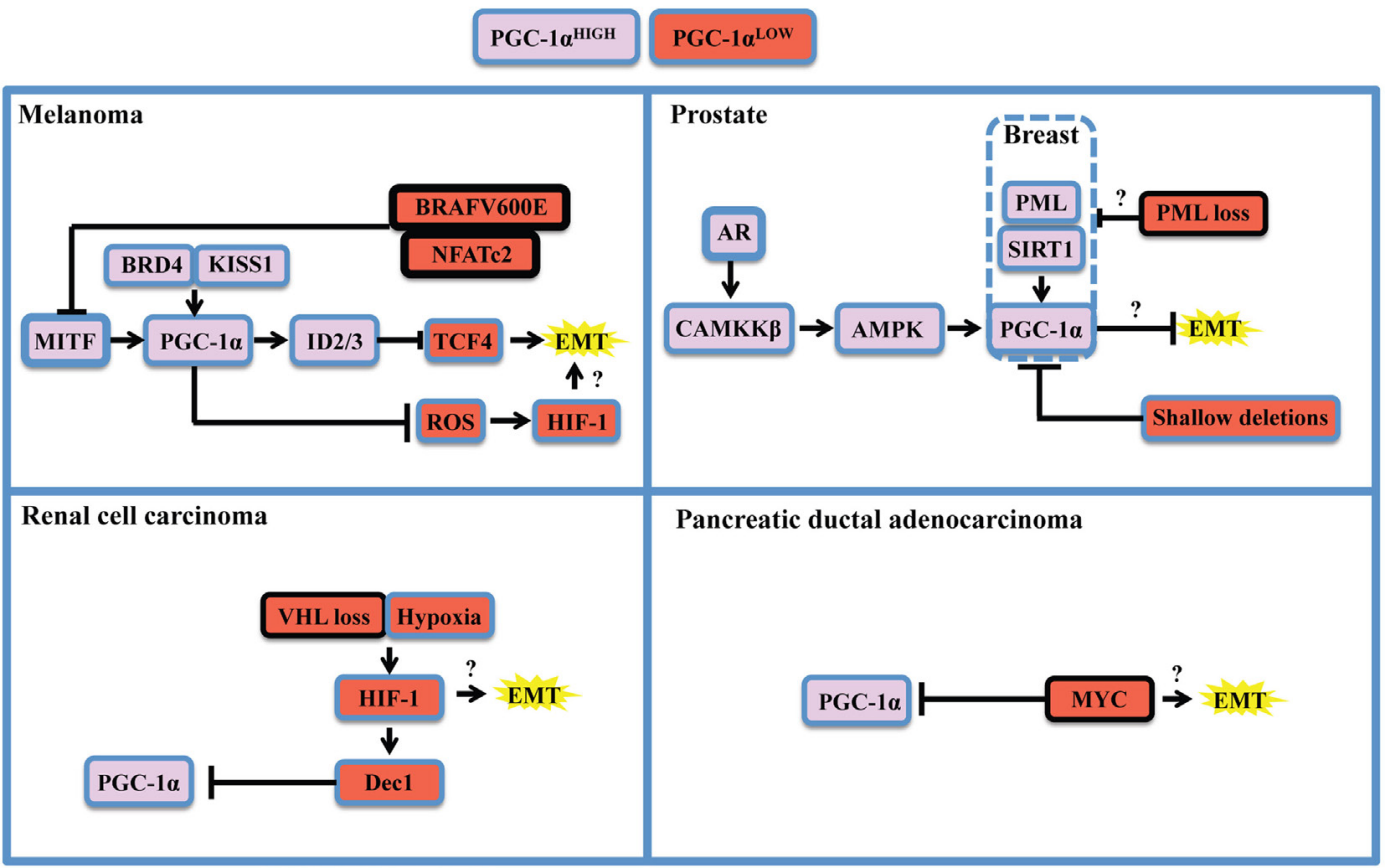
Oncogenic and context-specific determinants that modulate PGC-1α in cancer. The expression of PGC-1α is influenced by the presence of oncogenes, the loss or dysfunction of tumor suppressors, and the expression of lineage-specific regulators. Context-specific regulation depends on the expression of lineage-specific modulators, such as microphtalmia-associated transcription factor (MITF) in melanoma, or the expression of the androgen receptor (AR) in prostate cancer. It is also function of cellular stresses, such as limitation in nutrient supply, hypoxia, and redox state, and will depend on the activity of the SIRT1 deacetylase and the AMP kinase (AMPK) that will potentiate PGC-1α transactivating functions and might modify its gene expression programs. The oncogenic landscape [BRAF mutation, MYC amplification, VHL, or promyelocytic leukemia (PML) loss] will regulate PGC-1α expression at the transcriptional level, likely through direct binding of transcriptional inhibitors to the *PPARGC1A* promoter. Cumulating evidences support the notion that PGC-1α and mitochondrial OXPHOS are antagonistic to the establishment of the epithelial-to-mesenchymal transition (EMT). One mechanism supporting the inhibitory role of PGC-1α on EMT is through induction of Inhibitors of DNA binding (ID) proteins 2 and 3 that suppress TCF4, an important modulator of EMT. Therefore, oncogenic signaling could relieve EMT inhibition by downregulating PGC-1α and OXPHOS. By suppressing reactive oxygen species (ROS) though transcriptional regulation of antioxidant genes, PGC-1α exerts antagonistic effects on hypoxia-inducible factor 1α (HIF-1α). These findings converge to delineate the existence of critical rheostats opposing the expression of PGC-1α to the one of oncogenes and pro-glycolytic transcription factors such as HIF-1α. Question marks underline unknown links in the specified cancers in the context of PGC-1α modulation.

As stated above, the apparent dichotomous effects that PGC-1α has on many steps of tumorigenesis are mostly explained by context-specific considerations. Recent literature revealed that the expression of PGC-1α is under the tight control of oncogenes, tumor, and metastasis suppressors, as well as lineage-specific determinants (Figure 2). The study of melanoma provided important insights into both oncogenic and cell-lineage regulation of PGC-1α (Figure 2, top left). Microphtalmia-associated transcription factor (MITF), a transcription factor whose expression is largely restricted to the melanocytic lineage, has been shown to be amplified in 30% melanomas and required for survival of a subset of melanomas (63). MITF directly binds the PGC-1α promoter and regulates the expression of mitochondrial genes (37, 38). Interestingly, PGC-1α has been shown to regulate MITF expression in melanocytes (64), suggesting that a possible feed-forward mechanism might also occur in melanomas. Additional mechanisms by which PGC-1α is positively regulated in melanoma are through KISS1-mediated increase in protein stabilization (65) and through direct transcriptional regulation by BRD4 (66). In melanoma, the differential impact of PGC-1α on tumorigenesis appears to be associated with specific melanoma subsets. While PGC-1α is essential to support mitochondrial respiration and survival in MITF-expressing cells (38), PGC-1α can also block melanoma progression by at least two distinct mechanisms. First, PGC-1α can directly regulate the expression of inhibitors of DNA binding (ID) proteins 2 and 3 that suppress TCF4, a major regulator of EMT and metastatic programs (61). Second, PGC-1α and hypoxia-inducible factor 1α (HIF-1α) were shown to inversely correlate through a ROS-dependent mechanism (67). Given that the interplay between HIF-1α and PGC-1α is not fully elucidated (28, 68), and that HIF-1α has major functions in tumorigenesis and metastasis (69, 70), it would be important to determine how HIF-1α can influence the outcome of PGC-1α in other cancer models. Importantly, the MIFT-PGC-1α-ID2/3 axis in under the control of the BRAF (V600E) oncogene, the most frequent BRAF mutation in melanoma. Mechanistically, BRAF appears to inhibit MITF transcription through ERK activation (37). In addition, NFATc2, a transcription factor activated by oncogenic BRAF (71), was recently identified as a potent suppressor of MITF-PGC-1α in melanoma cells (72). Thus, multiples pathways under the control of BRAF might converge to oppose PGC-1α and OXPHOS in subsets of melanoma.

The study of PGC-1α in prostate cancer revealed a completely different regulatory landscape (Figure 2, top right). Prostate cancer is also a highly heterogeneous disease in which PGC-1α appear to have dualistic functions (36, 73). PGC-1α expression is lost during disease progression, notably by genomic deletions, which suppresses a gene expression program mediated by PGC-1α and by the nuclear receptor estrogen-relator receptor α (36). Besides, PGC-1α expression is enriched in a subset of prostate cancer patients in late disease stages (52). Importantly, two mechanisms that could potentially regulate PGC-1α in prostate cancer rely on AMP kinase (AMPK) and the sirtuin SIRT1. AMPK and SIRT1 are sensors of metabolic stress that will, respectively, phosphorylate and deacetylate PGC-1α in order to promote its transactivating functions (74). Strikingly, both proteins have been recognized for their dualistic contribution to cancer (75, 76), suggesting that the context-specific dualism of PGC-1α might correlate with AMPK or SIRT1 statuses. Mechanistically, the androgen receptor, which is required for prostatic cancer cell growth and survival, is able to modulate PGC-1α through CAMKKβ kinase and AMPK (52). Besides, the promyelocytic leukemia (PML) gene encodes for a tumor suppressor protein that potentiates PGC-1α deacetylation through SIRT1, thus promoting bioenergetics and protection against anoikis in breast cancer (77). Interestingly, PML is frequently co-deleted with PTEN in metastatic prostate cancer (78), suggesting that PML loss could modulate PGC-1α in prostate cancer through SIRT1. Moreover, PGC-1α mediates the pro-metastatic functions of SIRT1 in other cancers such as hepatocellular carcinoma (40), further strengthening the importance of the SIRT1-PGC-1α axis in cancer.

Finally, other PGC-1α regulatory modules have been described for renal cell carcinoma (Figure 2, bottom left) and pancreatic ductal carcinoma (Figure 2, bottom right). These studies revealed that PGC-1α expression can be downregulated by HIF-1α-mediated gene transcription of Dec1 (35) and by the MYC oncogene through direct binding of the *PPARGC1A* promoter (53). In both cases, the ratios of PGC-1α to HIF-1α or MYC function as rheostats that will dictate the propensity of cancer cells to rely on glycolysis or OXPHOS for survival. Hence, therapeutic strategies targeting the BRAF oncogene, HIF-1 α, or MYC will promote PGC-1α-dependent mitochondrial biogenesis and will favor OXPHOS addiction, thus revealing vulnerabilities to metabolic agents such as biguanides that target complex I of the electron transport chain (79). Moreover, recent studies unveiled additional determinants of PGC-1α expression in breast cancer. Mitochondrial mass and PGC-1α expression are controlled by β-catenin in estrogen receptor positive breast cancer cells (80). Interestingly, PGC-1α expression is higher in the HER2+ and basal breast cancer subtypes, both associated with lower survival (32). It would be relevant to further characterize the interplay between HER2 and the capacity of PGC-1α-expressing cells to metastasize, given pleiotropic roles of this oncogenic receptor in breast cancer metastasis (81–83).

## Conclusion

To summarize, it is now appreciated that diverse oncogenic and lineage- and context-specific determinants can potentially modulate PGC-1α expression and function in cancer. For a given cancer, assessment of PGC-1α status to predict outcome seems insufficient without an exhaustive documentation on these critical determinants. Here, we presented different key mechanisms that explain the dualist impact of PGC-1α on cancer progression. A key idea emerging from recent literature is that while PGC-1α and mitochondrial OXPHOS usually suppress the metastatic process, PGC-1α might still provide growth advantage *in vivo* by modulating other pathways in a context-dependent manner. Besides its regulation through sensing of various cellular stresses, its expression level and activity might be highly dynamic and heterogeneous during tumor evolution. Therefore, the survival of cancer cells and their ability to metastasize will be function of selective pressure dictated by the tumor microenvironment. Hence, systematic evaluation of oncogenes and context-specific determinants will undoubtedly contribute to expand our understanding of PGC-1α function within a given context and will pave the way to the development of new prognostic marker combinations and new therapeutic strategies.

## Author Contributions

S-PG wrote the manuscript, designed, and prepared the figures.

## Conflict of Interest Statement

The author declares that the research was conducted in the absence of any commercial or financial relationships that could be construed as a potential conflict of interest.
